# Combination of simvastatin, calcium silicate/gypsum, and gelatin and bone regeneration in rabbit calvarial defects

**DOI:** 10.1038/srep23422

**Published:** 2016-03-21

**Authors:** Jing Zhang, Huiming Wang, Jue Shi, Ying Wang, Kaichen Lai, Xianyan Yang, Xiaoyi Chen, Guoli Yang

**Affiliations:** 1 Department of Implantology, Stomatology Hospital, School of Medical, Zhejiang University, Yan’an Road, Hangzhou, P. R. China; 2 Department of Endodontics, Stomatology Hospital, School of Medical, Zhejiang University, Yan’an Road, Hangzhou, P. R. China; 3 Zhejiang-California International Nanosystem Institute, Zhejiang University, Hangzhou 310058, China; 4 Clinical Research Institute, Zhejiang Provincial People’s Hospital, No. 158 Shangtang Road, Hangzhou 310014, Zhejiang Province, China

## Abstract

The present study was performed to determine whether simvastatin improves bone regeneration when combined with calcium silicate/gypsum and gelatin (CS-GEL). The surface morphology was determined using field-emission scanning electron microscopy (FSEM). Degradation *in vitro* was evaluated by monitoring the weight change of the composites soaked in phosphate buffered saline (PBS). Drug release was evaluated using high-performance liquid chromatography (HPLC). Cytotoxicity testing was performed to assess the biocompatibility of composites. Four 5 mm-diameter bone defects were created in rabbit calvaria. Three sites were filled with CS-GEL, 0.5 mg simvastatin-loaded CS-GEL (SIM-0.5) and 1.0 mg simvastatin-loaded CS-GEL (SIM-1.0), respectively, and the fourth was left empty as the control group. Micro-computed tomography (micro-CT) and histological analysis were carried out at 4 and 12 weeks postoperatively. The composites all exhibited three-dimensional structures and showed the residue with nearly 80% after 4 weeks of immersion. Drug release was explosive on the first day and then the release rate remained stable. The composites did not induce any cytotoxicity. The results *in vivo* demonstrated that the new bone formation and the expressions of BMP-2, OC and type I collagen were improved in the simvastatin-loaded CS-GEL group. It was concluded that the simvastatin-loaded CS-GEL may improve bone regeneration.

Bone defects are often seen in clinical situations. Autografts, allografts, and artificial bone substitutes have been widely used in bone repair. Autografts have some specific drawbacks, such as the limited source material and risks of unpredictable resorption and morbidity at the donor site^1^. One alternative to autografts, allografts may transmit disease and induce immune responses if not pretreated appropriately^2^.

A variety of artificial bone substitutes are also being widely used in practice. These include metals, synthetic polymers such as poly lactic acid (PLA) and polyurethane (PU), and ceramics such as hydroxyapatite (HA) and β-tricalcium phosphate (β-TCP)^3^,^4^,^5^,^6^. Among the numerous bone substitutes, calcium sulfate dihydrate (CaSO_4_∙2H_2_O), called gypsum, has been used to repair bone defects for over 100 years^7^. Gypsum is formed by the hydration of calcium sulfate hemihydrate (CaSO_4_∙0.5H_2_O), which undergoes *in situ* setting after filling bone defects. Due to its considerable biocompatibility, gypsum has already been approved by the FDA for clinical use as a bone graft substitute^8^. However, because of the poor bioactivity of pure gypsum, it is difficult to form an effective chemical bond with the newly formed bone during the early healing stage^9^. It has been reported that composite biomaterials may be more suitable than pure biomaterials^10^. In a study by Wang, calcium silicate/gypsum was found to induce apatite formation on the surface of cement after incubation in simulated body fluid, which means the calcium silicate/gypsum composite has good bioactivity^11^. This shows that the addition of calcium silicate to form a composite biomaterial sidesteps the disadvantages of pure gypsum.

Gelatin has good biocompatibility and efficient hemostatic properties. It is also completely biodegradable *in vivo* and its physicochemical characteristics can be appropriately modulated^12^,^13^. Because of its strong adhesive and plasticity properties, gelatin can form a suitable matrix in calcium silicate/gypsum composites. An early study indicated the potential of the calcium sulfate and gelatin composite as a biodegradable bone substitute and in the promotion of new bone ingrowth^14^.

In order to increase the bioactivity of calcium silicate/gypsum and gelatin composite (CS-GEL), growth factors and drugs supporting bone regeneration should be incorporated into the composite. Simvastatin, a cholesterol-lowering drug, has been shown to have a positive effect on bone formation and bone mineral density *in vivo*^15^. Local application of simvastatin has been shown to promote fracture healing in ovariectomized rats^16^. Mukozawa *et al.* demonstrated that simvastatin with hydrogel and atelocollagen sponge (ACS) enhanced the bone growth of critical-sized nasal defects in rabbits^17^. Sukul *et al.* manufactured a simvastatin-loaded gelatin-nanofibrillar cellulose-beta tricalcium phosphate hydrogel scaffold, and reported that the scaffold could release the optimum concentration of simvastatin to enhance osteogenesis^18^. It has also been reported that the combination of simvastatin and gypsum can stimulate bone regeneration^19^. It has been demonstrated that gypsum has potential as a carrier for local release of antibiotics, growth factors, and drugs^20^,^21^,^22^.

All these findings show that the combination of simvastatin, calcium silicate/gypsum, and gelatin has promise as a bone substitute in the promotion of bone growth. In this study, the characteristics of this composite material were determined and its effects on the healing of calvarial defects in the rabbit were evaluated.

## Materials and Methods

### Powder preparation

For the preparation of bone substitute, simvastatin (Sigma-Aldrich, St. Louis, MO, U.S.), gelatin (Sigma-Aldrich, St. Louis, MO, U.S.) and calcium silicate/gypsum powders were donated by Professor Gou of the Bio-nanomaterials and Regenerative Medicine Research Division of Zhejiang University. The calcium silicate/gypsum powder consisted of 87.5% weight ratio (wt) of calcium sulfate hemihydrate (CaSO_4_·0.5H_2_O) and 12.5% wt of calcium silicate (CaSiO_3_). The preparations of calcium sulfate hemihydrate powder and calcium silicate powder have been described in Wang’s study^11^. In short, the calcium sulfate hemihydrate powder was made from calcium sulfate dehydrate (CaSO_4_·2H_2_O), which was treated in boiling 15% NaCl solution and stirred for 5 h at 100 °C with 0.1% citric acid. Then the suspension was filtered, washed three times in boiling water, and dried at 120 °C for 6 h. The calcium silicate powder was prepared using the chemical precipitation method: First, 500 ml Na_2_SiO_3_ solution (0.4 mol/L) was added to 500 ml Ca(NO_3_)_2_ solution (0.4 mol/L) dropwise with stirring; then the solution was filtered and washed three times each with deionized water and ethanol; last, the precipitates were dried at 80 °C for 24 h and calcined at 800 °C for 3 h.

### Preparation of CS-GEL and simvastatin-loaded CS-GEL

The CS-GEL composite was made of 0.05 g calcium silicate/gypsum powder, 0.01 g gelatin, and 100 μl deionized water, which was mixed thoroughly and then used to fill the bone defect. In order to compare the osteogenic effect of two doses of simvastatin in CS-GEL composite, 0.5 mg or 1.0 mg simvastatin replaced the equivalent calcium silicate/gypsum powder. First, simvastatin was mixed with gelatin to prepare drug-loaded gelatin, and then calcium silicate/gypsum powder was added to prepare simvastatin-loaded CS-GEL composite. To evaluate the structural characteristic, the CS-GEL composite or simvastatin-loaded CS-GEL composite was mixed with deionized water and then transferred to a circular mold to create cylinders. Then it was allowed to completely air-dry.

### Characterization of the composite

The longitudinal section morphology of the CS-GEL or simvastatin-loaded CS-GEL cylinders before and after incubation in PBS at 37 °C for 24 h was observed by field-emission scanning electron microscopy (FE-SEM, SINION-100, FEI). The surfaces of the cylinders after incubation in PBS for 24 h were further detected using energy dispersive x-ray spectroscopy (EDX). X-ray diffraction (XRD, X’Pert PRO, PANalytical) was used to determine the compositional analysis of CS-GEL and simvastatin-loaded CS-GEL at a scanning speed of 0.10°/min, Cu-Ka radiation (λ = 1.541 Å, 40 mA, 40 kV) at a 2θ range of 5–40°.

### Degradation *in vitro*

Degradation of the composites *in vitro* was evaluated by detecting the weight change of the cement samples soaked in 2 ml PBS at 37 °C. The soaking solution was refreshed every 2 days. At every set point, the samples were removed from the solution, rinsed with ethanol, and dried at 80 °C by weighing using an electronic analytical balance. The degradation was valued as the equation: Degradation (%) = weight after soaking/the initial weight × 100%.

### Measurement of simvastatin release from simvastatin-loaded CS-GEL

The two sets of simvastatin-loaded CS-GEL cylinders were soaked in 1.0 ml PBS solution in a sealed bottle at 37 °C, and the PBS was changed after 1 h, 3 h, 6 h, 1 d, 3 d, 7 d, 10 d, 14 d, and 21 d. At every set point, the simvastatin concentration of the soaking solution was collected. In view of the insolubility of simvastatin in PBS, the soaking solution was mixed with equal volume of methanol to dissolve simvastatin thoroughly. Then the solution was measured using high-performance liquid chromatography (HPLC, LC-20A, Shimadzu, JP) according to a standard curve prepared using specific amounts of simvastatin in advance.

### Cytotoxicity test

The cytotoxicities of the composites were evaluated using the extraction method, with mouse preosteoblast cells (MC3T3-E1). The cells were cultured in alpha-Minimum Essential Medium (*α*-MEM; Gibco, Tulsa, OK, U.S.) with 10% fetal bovine serum (FBS; Gibco). The extract solutions were prepared by immersing the composite pastes in 1.0 ml *α*-MEM for 24 h at 37 °C. The extracts were obtained and filtered through a 0.22 μm Millipore membrane. In order to observe a possible dose-dependant effect, the extract solution was diluted to 1/2, 1/4, 1/8, 1/16, 1/32, and 1/64 of the original concentration with *α*-MEM.

MC3T3-E1 cells were seeded into 96-well plate at 5 × 10^3^ cells per well and incubated for 24 h at 37 °C and 5% CO_2_ to allow the attachment of the cells. Then the culture medium was removed and replaced with 100 μl original extracts or diluted extracts with 10% FBS. The *α*-MEM containing 10% FBS and no extracts served as the control. After three days of culture, cell viability was evaluated using a cell-counting kit (CCK-8; Dojindo Molecular Technologies, Tokyo, Japan) according to the manufacturer’s instructions. After the indicated incubation times, the culture solutions were removed and 100 μl CCK-8 mixture solution was added to each well and incubated for 3 h at 37 °C. Optical density of each well was recorded at 450 nm using a multifunctional microplate reader (SpectraMax M5, Molecular Devices, U.S.). Cell viability was calculated as the percentage of the control group.

### Animal and surgical procedure

Sixteen male adult New Zealand rabbits (2.5–3.0 kg) were used in the study. The animals were kept in individual cages and fed and watered. The experiment was in line with the Institutional Animal Care and Use Committee of Zhejiang University, Hangzhou, China. The study protocol was reviewed and approved by the Ethics Committee for experimental animals, Zhejiang University (no. 866, Yuhangtang Road, Hangzhou, P.R. China) and in accordance with the ARRIVE guidelines^23^.

**Figure 1:**
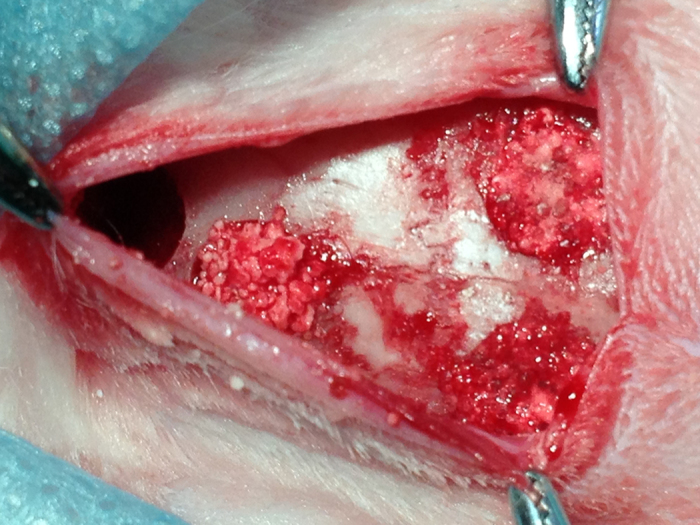
Four 5 mm-diameter bone defects in the rabbit calvarium: one empty defect served as the control group, and the other defects were filled with CS-GEL, SIM-0.5, and SIM-1.0, respectively.

Surgical procedures were performed under sterile conditions in a veterinary operating theater. Under general anesthesia induced by intramuscular injection of SuMianXin II (0.1 to 0.2 mL/kg, intramuscularly [IM], Quartermaster University of PLA, Changchun, China, Military Veterinary Institute), the areas of the scalp covering the calvarial vault were shaved and prepped with povidone iodine. After infiltration of local anesthesia (2% lidocaine with 1:100,000 epinephrine), an incision was made along the midline. Full-thickness skin and the periosteum were reflected to expose the cranium surface. Under saline irrigation, four circular defects, each 5 mm in diameter and 2 mm in depth, were created around the sagittal suture using a trephine bur. During drilling, great care was taken to avoid damage to the dura mater. For every animal, one defect each was filled with CS-GEL, 0.5 mg simvastatin-loaded CS-GEL (SIM-0.5), 1.0 mg simvastatin-loaded CS-GEL (SIM-1.0), or left untreated, respectively (Fig. 1). Lastly, surgical sites were sutured and the animals received antibiotics (penicillin, 400,000 U/d) for 3 days. At 4 and 12 weeks after surgery, the animals were euthanized by an overdose of SuMianXin (1.0 mL, IM).

### Micro-CT analysis

After the animals were euthanized, their calvarial bones containing CS-GEL or simvastatin-loaded CS-GEL were removed, fixed in 10% formaldehyde for 24 h. Three-dimensional microcomputed tomography imaging was performed on the specimens using a high-resolution microcomputed tomography system (SkyScan 1176; Bruker, Belgium) at a resolution of 17.46 μm. Specimens were scanned at 55 kV and 455 μA with a 0.5-mm aluminum filter and an exposure time of 250 milliseconds. Radiographic images were produced by the reconstruction software (Nrecon, SkyScan, DataViewer; Brucker, Kontich, Belgium). Analysis was performed using a 5 mm diameter circular region which was placed in the center of the initial defect area. The grey threshold was set in the range of 80–160 to avoid scan noise. The amount of regenerated bone tissue was obtained by analyzing bone volume fraction of the total tissue volume – bone volume/total volume (BV/TV).

### Specimen preparation and histological analysis

After micro-CT analysis, the specimens were decalcified in 0.5% formaldehyde containing 10% EDTA, at 4 °C for 5 weeks. After an adequate level of demineralization, the specimens were dehydrated in a graded alcohol series, embedded in paraffin, and sectioned into three pieces at 6 μm thickness. Sections were perpendicular to the sagittal suture near the defect central. One section was stained with hematoxylin and eosin (H&E). Images were acquired using a light microscope at ×40 and ×100 magnifications. New bone formation within the region bounded by the reversal lines was measured in three randomly selected fields in the area of the defect using an Image-Pro Plus software. The other two sections were immunohistochemically stained for histological analysis. Antibody to bone morphogenetic protein 2 (BMP-2) (bs-1012R, Bioss), antibody to osteocalcin (OC) (ab13418, Abcam) and antibody to type I collagen (AM10043SU-N, Acris Antibodies) were used as primary antibodies. The secondary antibody (k5007, Dako) was incubated at room temperature for 50 min. Immunohistochemical staining was performed on all slides to ensure the same antibody reaction would take place under the same diaminobenzidine staining conditions. Samples were imaged with a light microscope at ×100 magnification. The antigen reactivity of BMP-2, OC and type I collagen was automatically assessed (%) using image analysis software. A calibration procedure for the image analysis software was set before the morphometric analysis. The measurements were made 5 times by an author to confirm the reproducibility of the results. The mean percentage of the antigen reactivity served as the results.

### Statistical evaluation

Statistical analysis was conducted using SPSS 16.0 (SPSS, Chicago, IL, U.S.). All of the data were displayed as mean ± standard deviation (SD). The results of cytotoxicity testing were analyzed with one-way ANOVA. Two-way ANOVA analysis was performed to detect differences in the four groups in the *in vivo* experiment. Significant differences were evaluated using the LDS method in multiple comparisons. *P*-values below 0.05 were considered statistically significant.

## Results

### Surface morphology and characteristics

**Figure 2:**
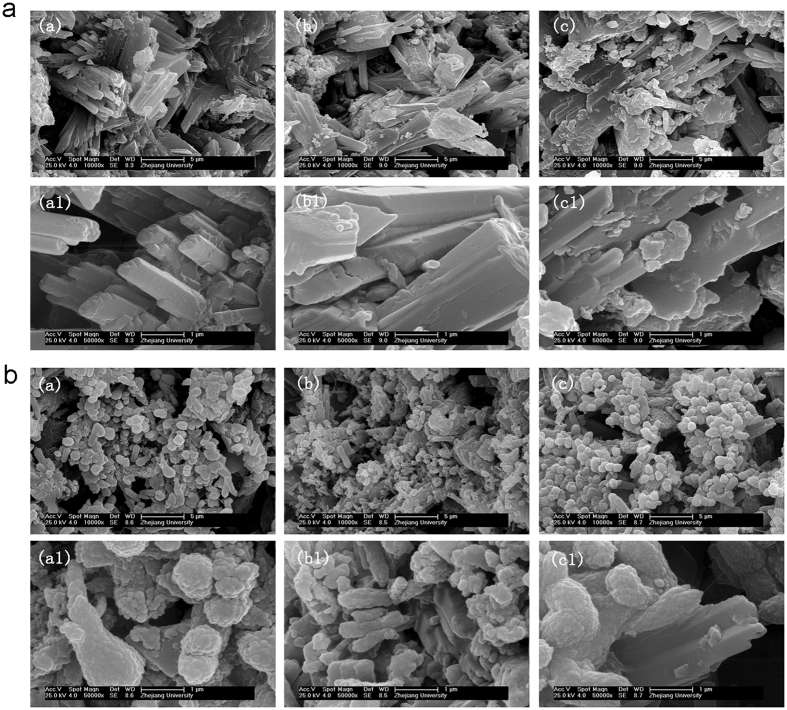
Surface morphology of the composites were determined by field-emission scanning electron microscopy (FSEM). high-performance liquid chromatography (HPLC). (**a**) SEM microphotograph of the longitudinal surface of the three types of composites. (a)(a1) CS-GEL; (b)(b1) SIM-0.5; (c)(c1) SIM-1.0. Original magnification a–c: ×10,000, and a1–c1: ×50,000. (**b**) SEM microphotograph of the longitudinal surface of the three types of composites after incubation in PBS for 24 h. (a)(a1) CS-GEL; (b)(b1) SIM-0.5; (c)(c1) SIM-1.0. Original magnification a–c: −10,000, and a1–c1: −50,000.

Microstructures of CS-GEL, SIM-0.5, and SIM-1.0 before and after incubation in PBS at 37 °C for 24 h are shown in Fig. 2a,b. The surface topographies in all groups exhibited a porous surface with a three-dimensional structure. When observed in detail, the three groups without incubation showed rod-like crystal structure of the calcium sulfate dihydrate with the calcium silicate aggregates distributed in the gypsum substrate. After incubation in PBS for 24 h, the majority of the crystals were covered with a layer of an apatite-like mineral, which was confirmed by the EDX analysis (Table 1). The results of EDX indicated that calcium phosphate salt may have been deposited on or absorbed onto the surface of the composites incubated in PBS and the Ca/P molar ratios were approximately 2.57 (26.91/10.46), 2.94 (20.78/7.08), and 2.75 (20.25/7.36) in CS-GEL, SIM-0.5, and SIM-1.0, respectively. There was also no obvious difference between the surface topography of CS-GEL, SIM-0.5, and SIM-1.0.

**Figure 3:**
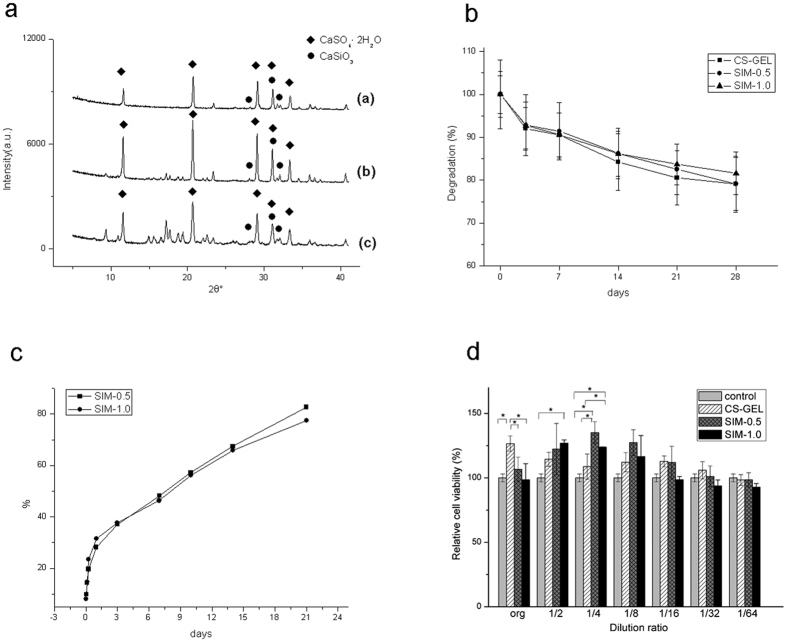
Physicochemical characteristics and cytotoxicities of the composites were evaluated. (**a**) X-ray diffraction (XRD) patterns of the three types of composites: (a) CS-GEL; (b) SIM-0.5; (c) SIM-1.0. (**b**) Degradation of the composites in PBS. (**c**) Cumulative release of simvastatin from SIM-0.5 and SIM-1.0. (**d**) Cell viabilities of MC3T3-E1 after exposure to the original and diluted extracts of the CS-GEL, SIM-0.5, and SIM-1.0 for three days (n = 3 in each group). *p < 0.05.

The XRD patterns of CS-GEL, SIM-0.5, and SIM-1.0 are shown in Fig. 3a. For the three groups, the peaks of CaSO_4_·2H_2_O and CaSiO_3_ were observed, which indicated that CaSO_4_·0.5H_2_O transformed to CaSO_4_·2H_2_O after the composite materials mixed with water.

### Biodegradation *in vitro*

As shown in Fig. 3b, the degradation of the CS-GEL and simvastatin-loaded CS-GEL cements after soaking in PBS for various time periods was evaluated. All the cements degraded rapidly in the first 3 days, and then the degradation rate decreased. After 21 days of immersion, the degradation became more stable. By day 28, there was no significant difference among the remaining weight of the three groups; however, the SIM-1.0 group showed the highest residue with over 80%.

### Release of simvastatin from samples

Drug release results of simvastatin are presented in Fig. 3c. Rapid simvastatin release was observed on the first day for the two groups. For the first day, 28.2% and 31.4% of simvastatin were released from SIM-0.5 and SIM-1.0, respectively. As the incubation time progressed, the simvastatin release rate decreased and a stable release was maintained. By day 21, 82.6% of the incorporated simvastatin had been released from SIM-0.5, and 77.5% had been released from SIM-1.0.

### Cytotoxicity assay

The viabilities of MC3T3-E1 cells after exposure to extracts of freshly mixed CS-GEL and simvastatin-loaded CS-GEL pastes for 3 days were assessed (Fig. 3d). For the original extracts, the CS-GEL group showed significantly higher cell viability than the other groups (F_(3,8)_ = 9.638, *p* = 0.005, one-way ANOVA. *p* = 0.002 vs the control group, *p* = 0.010 vs the SIM-0.5 group, *p* = 0.002 vs the SIM-1.0 group, LSD method), indicating the biocompatibility of the CS-GEL composite. For SIM-0.5 and SIM-1.0, the optical density values for the cells in the extracts diluted to 1/4 were significantly higher than those in the control group and the CS-GEL group (SIM-0.5: *p* < 0.001 vs the control group, *p* = 0.001 vs the CS-GEL group, SIM-1.0: *p* = 0.002 vs the control group, *p* = 0.024 vs the CS-GEL group, LSD method). For SIM-1.0, the optical density values for the cells in the extracts diluted to 1/2 were significantly higher than that in the control group (*p* = 0.008, LSD method). The cell viability of the extracts diluted from 1/8 to 1/64 did not show significant differences among the four groups. (1/8 dilution: F_(3,8)_ = 3.795, *p* = 0.058, 1/16 dilution: F_(3,8)_ = 3.734, *p* = 0.06, 1/32 dilution: F_(3,8)_ = 2.139, *p* = 0.173, 1/64 dilution: F_(3,8)_ = 1.605, *p* = 0.263, one-way ANOVA). The SIM-0.5 and SIM-1.0 extracts diluted to 1/4 showed significantly higher cell viability than the corresponding original extracts and extracts diluted to 1/32 and 1/64 (SIM-0.5: F_(3,8)_ = 13.330, *p* = 0.002, SIM-1.0: F_(3,8)_ = 53.097, *p* < 0.001, one-way ANOVA).

### Micro-CT analysis

**Figure 4:**
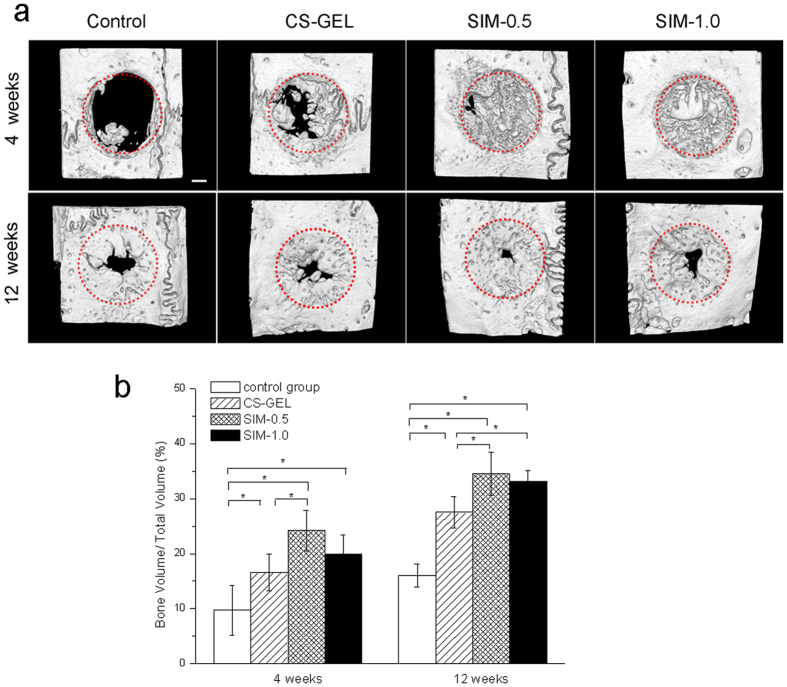
Microcomputed tomography (Micro-CT) analysis of the new bone formation at 4 and 12 weeks. (**a**) Representative micro-CT scans showing the bone regeneration in the defects after 4 weeks and 12 weeks. The red circles indicate the edges of the defect. Scale bar, 1 mm. (**b**) Multiple-comparison analysis of regenerated bone volume fraction in defects (BV/TV) treated with different groups (n = 3 in each group) at each experimental point in time. *p < 0.05.

As shown in Fig. 4a, the defect coverage was minimal in the control group at 4 weeks of healing, and almost all defect edges remained when compared with other groups. In contrast, the CS-GEL group, simvastatin-loaded CS-GEL groups demonstrated remarkable mineralized tissue formation within the defect area. At 12 weeks, the amount of new bone formation in all groups increased markedly, which appeared to be synthesized towards periphery of the defect.

The bone volume fraction of the total tissue volume (BV/TV) has been analysed to evaluate the amount of new bone regeneration (Fig. 4b). Results of the ANOVA test exhibited that the values of BV/TV differed significantly among the four groups (4 weeks: F_(3,6)_ = 10.468, *p* = 0.008, 12 weeks: F_(3,6)_ = 40.958, *p* < 0.001, two-way ANOVA). The SIM-0.5 group exhibited significantly greater values for BV/TV than the control group and CS-GEL group at both 4 weeks and 12 weeks (4 weeks: *p* = 0.002 vs control group, *p* = 0.029 vs CS-GEL group, 12 weeks: *p* < 0.001 vs control group, *p* = 0.006 vs CS-GEL group, LSD method). The values for BV/TV in the CS-GEL group and SIM-1.0 group were significantly higher than in the control group at 4 and 12 weeks (4 weeks: *p* = 0.042 vs CS-GEL group, *p* = 0.009 vs SIM-1.0 group, 12 weeks: *p* = 0.001 vs CS-GEL group, *p* < 0.001 vs SIM-1.0 group, LSD method).

### Histomorphometric analysis

**Figure 5:**
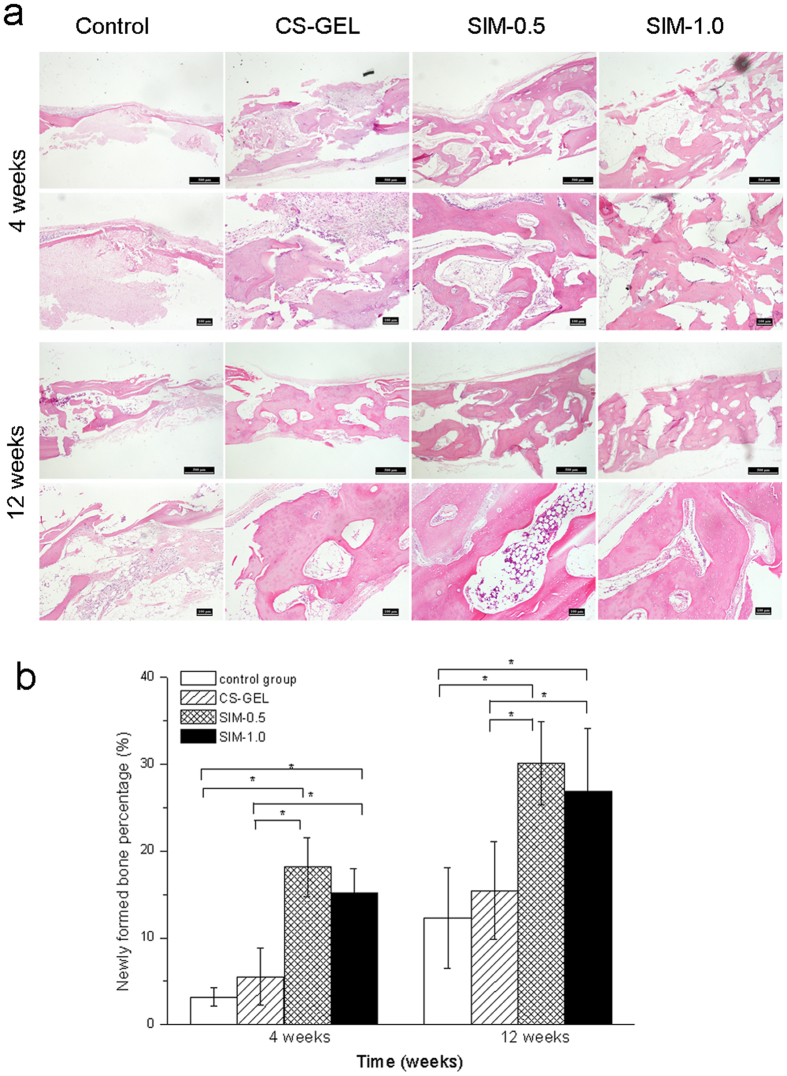
Histological analysis of the new bone formation at 4 and 12 weeks. (**a**) Representative views of hematoxylin and eosin staining of calvarial bone at 4 and 12 weeks. Original magnification, 1^st^ and 3^rd^ rows: ×40, 2^nd^ and 4^th^ rows: ×100. (**b**) Multiple-comparison analysis of newly formed bone percentage in rabbit calvarial defect between different groups (n = 8 in each group) at each experimental point in time using the LSD method. *p < 0.05.

At 4 weeks, a large amount of eosin-stained newly formed bone, characterized by the irregular trabeculae of immature bone and osteoid rimmed by osteoblasts, was observed in the defects of SIM-0.5 and SIM-1.0 (Fig. 5a). Moreover, some of the composite materials remained in the defect area, which were surrounded by new bone tissues. Less newly formed bone was observed in the CS-GEL group. In contrast, most areas of the defects in the control group were filled with fibrous connective tissue. In the 12th week, the bone tissues in the defect area in SIM-0.5 and SIM-1.0 were completely ossified and similar to normal bone in appearance (Fig. 5a). Newly formed marrow cavities were also observed.

At 4 weeks, histomorphometric analysis showed the percentage of the newly formed bone to be 3.19 ± 1.08% of the area of interest in the control group, 5.53 ± 3.30% in CS-GEL, 18.16 ± 3.40% in SIM-0.5, and 15.19 ± 2.78% in SIM-1.0 (Fig. 5b). At 12 weeks, newly formed bone occupied 12.29 ± 5.82% of the area of interest in the control group, 15.44 ± 5.65% in the CS-GEL group, 30.13 ± 4.80% in the SIM-0.5 group, and 26.87 ± 7.21% in the SIM-1.0 group (Fig. 5b). The results suggested that simvastatin-loaded CS-GEL induced the remarkable improvement of new bone formation (4 weeks: F_(3,21)_ = 50.829, *p* < 0.001, 12 weeks: F_(3,21)_ = 57.155, *p* < 0.001, two-way ANOVA). The *post hoc* analysis exhibited that the values of new bone formation in SIM-0.5 and SIM-1.0 were significantly higher than in other groups at both 4 and 12 weeks (*p* < 0.001, LSD method).

### Expression of BMP-2, OC and type I collagen

**Figure 6:**
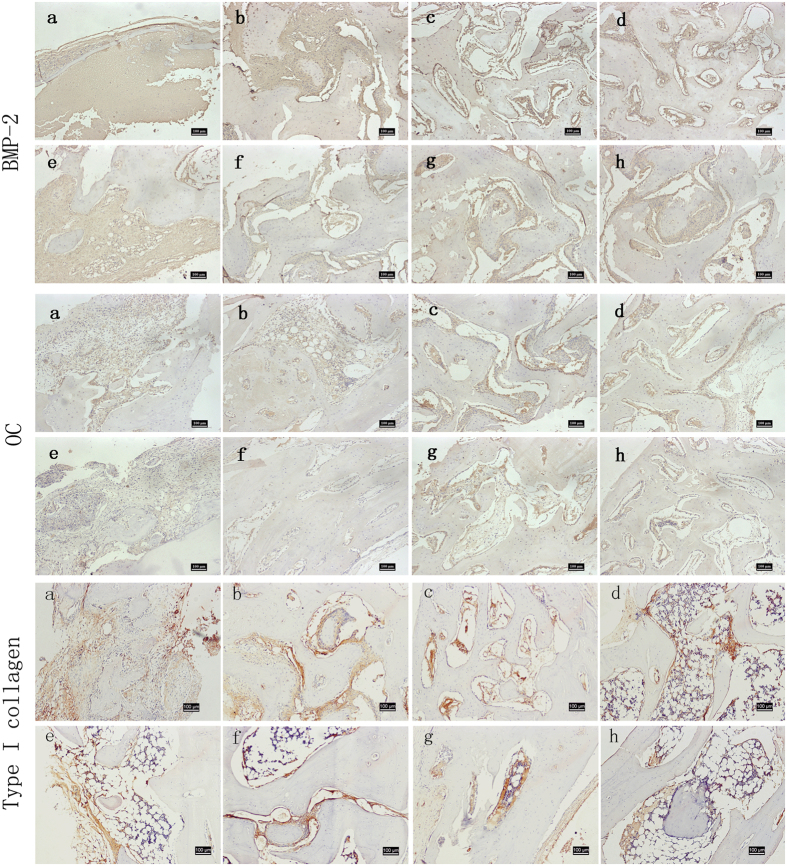
Representative views of immunohistochemical staining for BMP-2, OC and type I collagen at (**a**–**d**) 4 weeks and (**e**–**h**) 12 weeks. **(a,e)**: control group, **(b,f)**: CS-GEL, **(c,g)**: SIM-0.5, **(d,h)**: SIM-1.0. Original magnification, ×100

**Figure 7:**
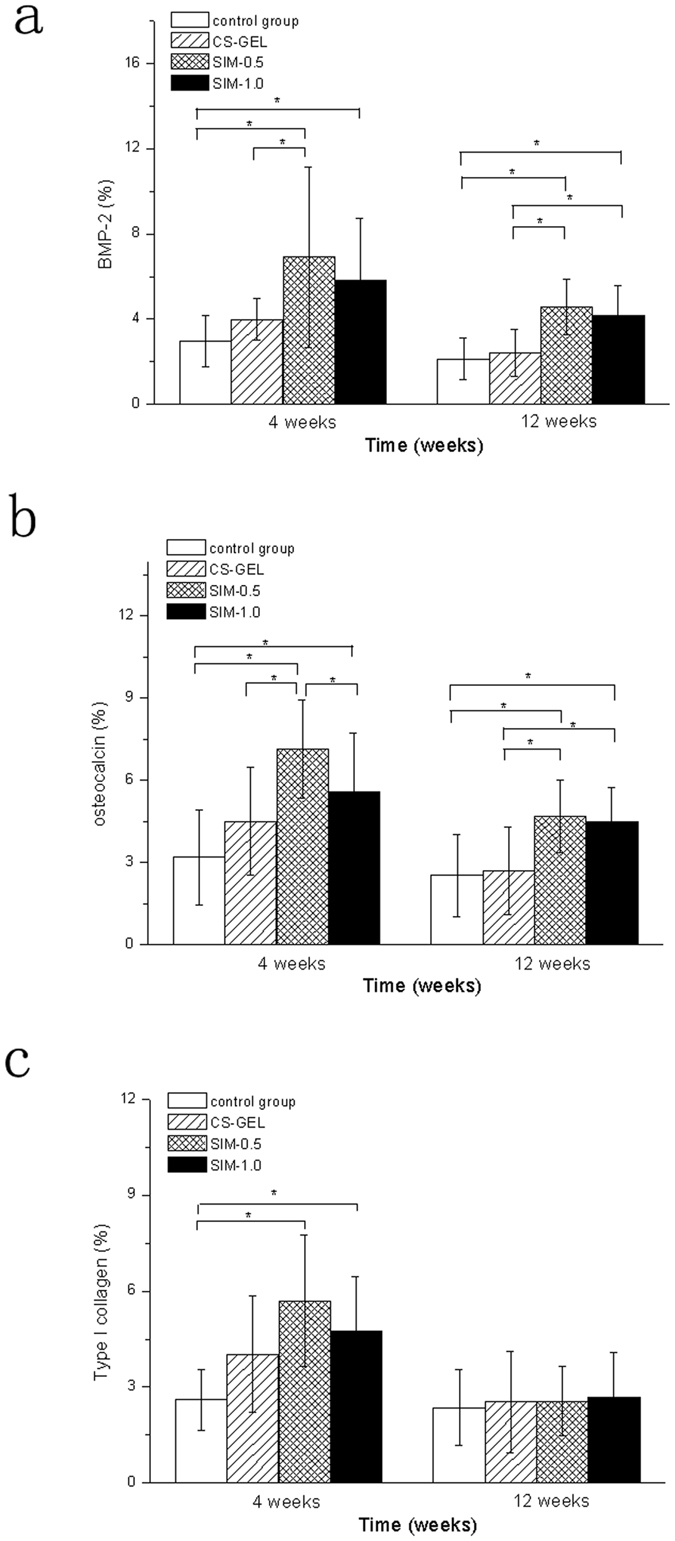
Multiple-comparison of histomorphometric analysis. (**a**) Multiple-comparison analysis of BMP-2 antigen reactivity in rabbit calvarial defect between different groups (n = 8 in each group) at each experimental point in time using the LSD method. *p < 0.05. (**b**) Multiple-comparison analysis of OC antigen reactivity in rabbit calvarial defect between different groups (n = 8 in each group) at each experimental point in time using the LSD method. *p < 0.05. (**c**) Multiple-comparison analysis of type I collagen antigen reactivity in rabbit calvarial defect between different groups (n = 8 in each group) at each experimental point in time using the LSD method. *p < 0.05.

The immunohistochemical staining of BMP-2, OC and type I collagen is shown in Fig. 6. At 4 and 12 weeks, significantly higher levels of positive expression of BMP-2 and OC were observed in SIM-0.5 than in the control group or CS-GEL group (Fig. 7a, 4 weeks: *p* = 0.001 vs the control group, *p* = 0.006 vs the CS-GEL group, 12 weeks: *p* = 0.001 vs the control group, *p* = 0.002 vs the CS-GEL group, LSD method. Figure 7b, 4 weeks: *p* < 0.001 vs the control group, *p* = 0.001 vs the CS-GEL group, 12 weeks: *p* = 0.011 vs the control group, *p* = 0.019 vs the CS-GEL group, LSD method.). The immunostaining values for BMP-2 and OC in SIM-1.0 were higher than those in the control group at 4 and 12 weeks with significant difference (Fig. 7a, 4 weeks: *p* = 0.008, 12 weeks: *p* = 0.003, LSD method. Figure 7b, 4 weeks: *p* = 0.002, 12 weeks: *p* = 0.020, LSD method.). When compared to CS-GEL, the BMP-2 and OC expressions in SIM-1.0 were significantly higher, but only at 12 weeks (Fig. 7a, *p* = 0.008, LSD method. Figure 7b, *p* = 0.032, LSD method). The antigen reactivity of type I collagen in SIM-0.5 and SIM-1.0 was higher than those in the control group at 4 weeks (Fig. 7c, 4 weeks: *p* = 0.003 vs SIM-0.5, *p* = 0.029 vs SIM-1.0, LSD method.). Moreover, the immunostaining value for OC in SIM-0.5 was significantly greater than in SIM-1.0 at 4 weeks (Fig. 7b, *p* = 0.029, LSD method). Additionally, the values of new bone formation and BMP-2 antigen reactivity in SIM-0.5 tended to be more pronounced than in SIM-1.0 at 4 and 12 weeks. The values of type I collagen antigen reactivity in SIM-0.5 were greater than in CS-GEL and SIM-1.0 at 4 weeks. However, these differences did not reach statistical significance (Fig. 5b, 4 weeks: *p* = 0.053, 12 weeks: *p* = 0.057, LSD method. Figure 7a, 4 weeks: *p* = 0.272, 12 weeks: *p* = 0.506, LSD method. Figure 7c, 4 weeks: *p* = 0.080 vs CS-GEL, *p* = 0.302 vs SIM-1.0, LSD method).

## Discussion

In this study, a combination of calcium silicate/gypsum and gelatin for use as bone substitute was developed and used to release simvastatin in a sustainable manner. Results demonstrated that simvastatin was released from simvastatin-loaded CS-GEL efficiently and simvastatin-loaded CS-GEL promoted new bone formation in rabbit calvarial defects.

An osteogenic effect was observed when a daily simvastatin dose of 5 mg/kg was given systemically by intraperitoneal^24^ or oral administration^25^. It is reported that simvastatin induces osteoblast differentiation via the BMP-2 pathway^26^ and exhibits anti-inflammatory effects^27^. However, simvastatin undergoes extensive hepatic extraction, which leads to a decrease in peripheral concentrations of statin, systemic application of simvastatin at a recommended dosage for hypercholesterolemia is insufficient for osteogenesis around implants^24^. Local application of simvastatin can bypass hepatic degradation to produce an osteogenic effect in bone and prevent side effects, including myotoxicity, liver failure, and kidney failure^28^,^29^,^30^.

Several studies have demonstrated the bone-promoting effect of local use of simvastatin with different carriers in various animal models. Nyan *et al.* observed remarkable bone formation in rat calvarial defects treated with a combination of 1.0 mg simvastatin and calcium sulfate, which suggests the potential of calcium sulfate as a carrier for the local release of simvastatin^31^. However, pure gypsum has a rapid degradation rate in biomimetic media which is adverse for matrix deposition and tissue growth. A study by Wang showed that the addition of calcium silicate into gypsum cement could lower its degradation rate and induce apatite remineralization and deposition on the composite surface^11^. In the study, calcium-silicate-doped gypsum was used instead of pure gypsum. Gelatin is highly biocompatible, bioresorbable, and can be made to fit defects of almost any shape because of its sponge-like form^32^. Gao *et al.* reported that the calcium sulfate/gelatin composite could promote new bone ingrowth^14^. In this way, the combination of calcium silicate/gypsum and gelatin seems to be an effective bone substitute and a carrier of simvastatin. In previous studies, the following doses of simvastatin used were investigated: 0.5 mg^32^,^33^, 2 mg^34^, 2.2 mg^35^, 0.1, 0.5, 1.0, 1.5 and 2.2 mg^36^. In this study, 0.5 mg and 1.0 mg simvastatin were used with the CS-GEL composite for calvarial bone regeneration.

The results indicated that CS-GEL and simvastatin-loaded CS-GEL have three-dimensional structures and considerable apatite deposition abilities. The deposition of calcium phosphate salt could promote protein adsorption and the attachment, proliferation, and differentiation of osteogenic cells^37^. It is possible that the lack of visible morphologic differences between CS-GEL and simvastatin-loaded CS-GEL was due to the low concentration of the incorporated simvastatin. The peak of CaSO_4_·2H_2_O was detected in the XRD patterns of CS-GEL and simvastatin-loaded CS-GEL. CaSO_4_·2H_2_O can be absorbed into the body, forming micropores in the implanted material, which facilitates the ingrowth of new bone tissue^38^. However, the property of rapid dissolution renders gypsum with limited applications in orthopedic and dental surgery. The rapid degradation rate adverses to the deposition of the bone-like apatite on the cement surface^39^. Results of the degradation test in the study provided the evidences that the simvastatin-loaded CS-GEL shared the similar degradation property with CS-GEL, and the degradation of the composites could last more than 4 weeks. In the release assay, the two concentrations of simvastatin-loaded CS-GEL groups showed highly efficient release of simvastatin on the first day, and the release of simvastatin in CS-GEL could last at least 3 weeks. We hypothesize that the simvastatin is released gradually from the degrading CS-GEL composites, which then could promote osteoblast differentiation and recruitment into the bone defect area.

The cytotoxicity assay indicated that CS-GEL, SIM-0.5, and SIM-1.0 did not induce any cytotoxicity. It was here noted that the cell viability in the original extracts from CS-GEL was higher than that in the other diluted extracts, which might be attributable to the higher concentrations of Ca and Si ions. Ca ions are essential to maintenance of the growth and function of living cells and they have a positive effect on the proliferation and differentiation of osteoblasts^40^. An appropriate concentration of Si ion has been shown to increase the proliferation, differentiation, and collagen production of osteoblasts^41^,^42^. For SIM-0.5 and SIM-1.0 samples, the viability of cells in the extracts diluted to 1/4 was greater than those in the corresponding original extracts and extracts diluted to 1/32 and 1/64, which indicated that the proper concentrations of simvastatin in the extracts may have positive effects on cell proliferation.

In this study, a rabbit calvarial defect model was used to test the hypothesis because it is a convenient model for research into bone regenerative materials and it does not have fixation requirements. As shown in the results described above, maximum bone regeneration was consistently observed in the defects treated with SIM-0.5. The results of micro-CT imaging analysis indicated that the simvastatin-loaded CS-GEL could facilitate the bone defect closure. In the histological sections of the 4 and 12-week samples, significantly more new bone formation and positive expression of BMP-2 and OC was observed in the SIM-0.5 group than in the control group and the CS-GEL group. The results of new bone fomation evaluated with histological examinations were basically consistent with micro-CT analysis. Despite the lack of statistical significance, the values of newly formed bone and BMP-2 antigen reactivity in SIM-0.5 tended to be more pronounced than in SIM-1.0 at 4 and 12 weeks. The expressions of BMP-2, OC, and type I collagen at 4 weeks were higher than those at 12 weeks, which indicates that BMP-2, OC, and type I collagen are mainly expressed at early stages of bone healing and decrease during the maturation process. Although local application of simvastatin could elicit inflammation, there was no obvious soft tissue inflammation in the simvastatin-loaded CS-GEL groups at 4 and 12 weeks in this study, which indicated that the doses of 0.5 mg and 1.0 mg simvastatin incorporated into CS-GEL could increase bone regeneration without inducing intense soft tissue inflammation.

In a study by Wong *et al.*, 0.5 mg simvastatin was added to a collagen matrix in rabbit calvarial defects, and a total of 308% more new bone was present in the simvastatin-collagen group than in the collagen group alone on postoperative day 14^33^. Huang *et al.* found no significant difference in bone formation between groups with rhBMP-2-loaded calcium sulfate and simvastatin-loaded calcium sulfate in rabbit bone defects. This indicated that the osteogenic effects of simvastatin are comparable to those of rhBMP-2^43^. Various studies have also investigated local administration of statins to promote bone healing and demonstrated the beneficial effects of locally applied statins for osteogenesis^31^,^35^,^36^,^44^,^45^. These results are consistent with those of the present study. However, some studies have shown adverse effects. Anbinder *et al.* failed to find any clear improvement in bone regeneration in rat tibial defects by using simvastatin, either orally or subcutaneously^46^. Similarly, the authors reported that the use of simvastatin and polymer scaffold, associated or not, did not improve bone regeneration^47^. This difference may be due to the experimental model, the means of administration of simvastatin, or the variable carriers and doses of simvastatin.

In the current study, only two concentrations of simvastatin in CS-GEL composite were evaluated, and more different concentrations of simvastatin should be tested in the experimental protocol. Further studies are required to identify the optimal bone substitutes and analyze the mechanisms underlying the promotion of bone regeneration by simvastatin.

In conclusions, the combination of calcium silicate/gypsum and gelatin was successfully used as a carrier for simvastatin. The results of *in vivo* experimentation indicated that the simvastatin-loaded CS-GEL may improve bone regeneration.

## Additional Information

**How to cite this article**: Zhang, J. *et al.* Combination of simvastatin, calcium silicate/gypsum, and gelatin and bone regeneration in rabbit calvarial defects. *Sci. Rep.* **6**, 23422; doi: 10.1038/srep23422 (2016).

## Figures and Tables

**Table 1:** Relative content of Ca, S, P, Si, and O on the surface of the composites as indicated by EDX analysis.

Atomic percent	Ca	S	P	Si	O
CS-GEL	26.91	2.3	10.46	2.09	58.23
SIM-0.5	20.78	2.22	7.08	6.54	63.37
SIM-1.0	20.25	3.33	7.36	8.26	60.81
